# phot1 Inhibition of ABCB19 Primes Lateral Auxin Fluxes in the Shoot Apex Required For Phototropism

**DOI:** 10.1371/journal.pbio.1001076

**Published:** 2011-06-07

**Authors:** John M. Christie, Haibing Yang, Gregory L. Richter, Stuart Sullivan, Catriona E. Thomson, Jinshan Lin, Boosaree Titapiwatanakun, Margaret Ennis, Eirini Kaiserli, Ok Ran Lee, Jiri Adamec, Wendy A. Peer, Angus S. Murphy

**Affiliations:** 1 Institute of Molecular Cell and Systems Biology, University of Glasgow, Glasgow, United Kingdom; 2 Department of Horticulture, Purdue University, West Lafayette, Indiana, United States of America; 3 Bindley Bioscience Center, Purdue University, West Lafayette, Indiana, United States of America; Cambridge University, United Kingdom

## Abstract

It is well accepted that lateral redistribution of the phytohormone auxin underlies the bending of plant organs towards light. In monocots, photoreception occurs at the shoot tip above the region of differential growth. Despite more than a century of research, it is still unresolved how light regulates auxin distribution and where this occurs in dicots. Here, we establish a system in *Arabidopsis thaliana* to study hypocotyl phototropism in the absence of developmental events associated with seedling photomorphogenesis. We show that auxin redistribution to the epidermal sites of action occurs at and above the hypocotyl apex, not at the elongation zone. Within this region, we identify the auxin efflux transporter ATP-BINDING CASSETTE B19 (ABCB19) as a substrate target for the photoreceptor kinase PHOTOTROPIN 1 (phot1). Heterologous expression and physiological analyses indicate that phosphorylation of ABCB19 by phot1 inhibits its efflux activity, thereby increasing auxin levels in and above the hypocotyl apex to halt vertical growth and prime lateral fluxes that are subsequently channeled to the elongation zone by PIN-FORMED 3 (PIN3). Together, these results provide new insights into the roles of ABCB19 and PIN3 in establishing phototropic curvatures and demonstrate that the proximity of light perception and differential phototropic growth is conserved in angiosperms.

## Author Summary

Plants depend on sunlight for photosynthesis and adapt their growth to optimize light capture. Phototropism, the reorientation of growth towards light, is one important adaptive response. Modern studies of phototropism began with experiments in monocotyledonous grasses by Charles Darwin and led ultimately to the discovery of the plant growth hormone auxin, establishing the concept that light perception at the shoot apex triggers differential bending in the tissues below. In the past two decades, molecular-genetic analysis in the model flowering plant *Arabidopsis thaliana* has identified the principle photoreceptor for phototropism, phot1, as well as the major auxin transporters. Despite extensive efforts, how the photoreceptor regulates auxin transport so as to establish differential growth is still poorly understood, as is whether this process is conserved between monocots and dicots. Here, we introduce a new approach to the study of *Arabidopsis* phototropism in the absence of developmental events associated with seedling photomorphogenesis. In doing so, we show that the proximity of light perception and differential growth is conserved between monocots and dicots: in both plant types, differential growth is a consequence of lateral auxin movements across the shoot apex. Moreover, we identify two auxin transporters, PIN3 and ABCB19, that contribute to these movements, the latter serving to prime lateral auxin fluxes in the shoot apex. ABCB19 function is regulated by phot1, identifying it as a substrate for this class of photoreceptor kinase.

## Introduction

Plants have evolved numerous ways to optimize photosynthetic light capture. Phototropism, the reorientation of growth towards light, is one of the most important of these adaptive processes [1]. Originally identified in monocot coleoptiles by Charles and Francis Darwin, phototropism is initiated by light perceived at the shoot tip generating a diffusible signal that influences differential elongation in the tissues below [2]. Subsequent studies have since shown that phototropism arises from increased growth on the shaded side of the stem [3], owing to an accumulation of the phytohormone auxin [4]. The role of PHOTOTROPIN (phot) blue-light receptors in establishing phototropic curvatures in the model dicot *Arabidopsis thaliana* is well established [5], as is evidence supporting auxin accumulation on the shaded side of photostimulated hypocotyls [6],[7].

To date, little is known regarding the molecular events that transduce photoreceptor activation into auxin redistribution across the growing stem. Genetic analyses in *Arabidopsis* have identified several auxin transporter families [4]. Of these, members of the PIN-FORMED family, named after the influorescence phenotype of the *pin1* mutant, are the primary mediators of directional auxin fluxes that regulate plant development [8]. PIN3 is proposed to mediate lateral auxin fluxes by differentially restricting auxin to the vascular cylinder [6]. Consistent with this mode of action, PIN3 exhibits a subcellular localization on the inner side of bundle sheath cells [6]. However, PIN3 also functions in apical hook opening [9], which may contribute to the delayed phototropism observed in etiolated *pin3* seedlings [6],[10].

Another transporter implicated in phototropism is ATP-BINDING CASSETTE B19 (ABCB19/MDR1/PGP19; herein abbreviated B19), which transports auxin out of the shoot apex and maintains long-distance auxin transport streams primarily by preventing cellular reuptake and diffusion into cells adjoining vascular tissues [11],[12]. B19 is predominantly localized apolarly at the plasma membrane [12]–[14] and functions coordinately with PIN1 to mediate polar auxin flow from the shoot apex to the roots [15]. Additionally, *b19* mutants exhibit enhanced phototropic curvature [10],[15] that has been attributed to auxin accumulation in the hypocotyl elongation zone as a consequence of decreased PIN1-B19-mediated polar transport [10],[15]. A role for B19 in seedling photomorphogenesis has also been reported that is dependent on CRYPTOCHROME (cry) and PHYTOCHROME (phy) photoreceptors [16],[17], both of which are known to impact phototropism in *Arabidopsis* [18],[19].

We therefore sought to clarify the roles of B19 and PIN3 in establishing phototropic curvatures by discriminating auxin transport processes associated with phototropism from those involved in seedling photomorphogenesis. To address this, we employed an unconventional strategy to analyze phototropic responses in *Arabidopsis* post-photomorphogenesis by subjecting light-grown seedlings to dark acclimation. By adopting this revised experimental approach, we uncover new mechanistic information regarding the roles of B19 and PIN3 in effecting phototropism and demonstrate a direct link between photoreceptor activation and auxin transporter function. Furthermore, we provide clear evidence that lateral auxin fluxes required for phototropism in *Arabidopsis* seedlings are initiated in and above the hypocotyl apex and not in the region of differential growth, analogous to the auxin transport mechanisms proposed for monocot phototropism almost a century ago.

## Results

### Hypocotyl Phototropism in *Arabidopsis* Following Dark Acclimation

Phototropism to directional blue light is traditionally examined using etiolated seedlings. However, studies performed in this manner examine the combined processes of phototropism and seedling photomorphogenesis. For the experiments described here, it was desirable to discriminate auxin transport processes associated with phototropism from those involved in seedling photomorphogenesis. To isolate phototropic responses from de-etiolation events, 3-d-old light-grown seedlings were subjected to a 24-h period of dark acclimation prior to phototropic stimulation.

**Figure 1:**
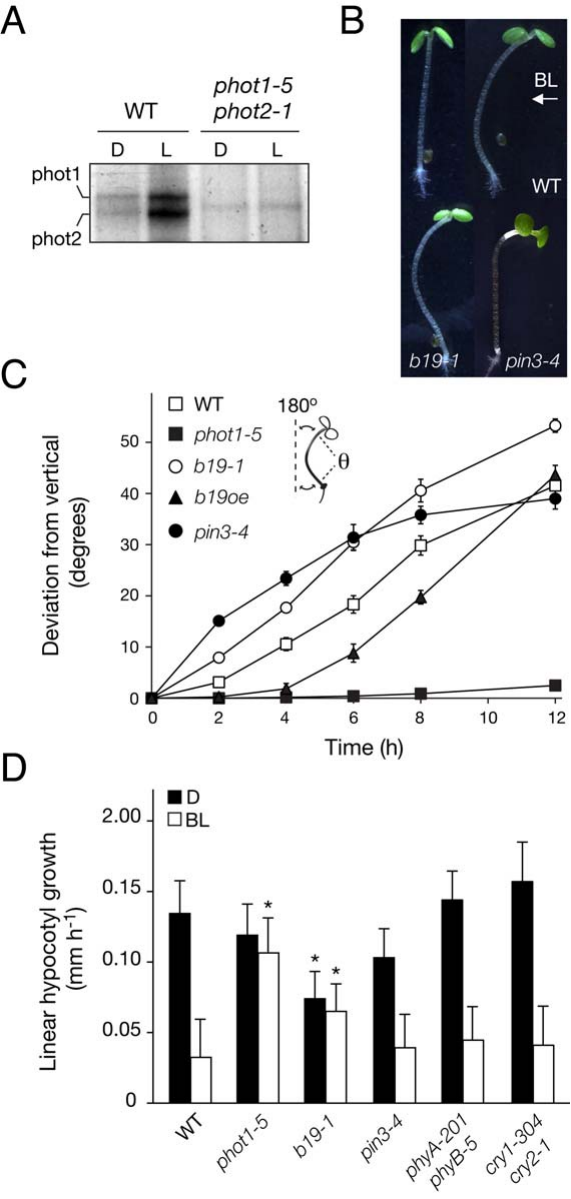
Phototropic response of dark-acclimated *Arabidopsis*. (A) Autophosphorylation activities for phot1 and phot2 in membranes isolated from wild-type (WT) and *phot1-5 phot2-1* seedlings. Protein extracts were given a mock irradiation (darkness, D) or irradiated with 10,000 µmol m^−2^ white light (L) in the presence of radiolabeled ATP. (B) Phototropic response of seedlings (upper left) exposed to directional blue light (BL, 1 µmol m^−2^ s^−1^) for 12 h (upper right). Also shown is the phototropic bending of *b19–1* (lower left) and *pin3–4* (lower right) to blue light for 12 h and 24 h, respectively. (C) Phototropic time course for wild-type, *phot1–5*, *b19–1*, *B19* overexpressing (*b19oe*), and *pin3–4* seedlings. Hypocotyl deviation from the vertical was calculated (inset) by subtracting the measured angle (θ) from the vertical to determine the total angle of deviation (indicated by the dotted lines). Results represent the mean + standard error, *n  = *10 seedlings. (D) Vertical hypocotyl growth within the first hour after treatment with directional blue light (1 µmol m^−2^ s^−1^) or continued darkness. Results represent the mean ± SD, *n  = *10 seedlings in three independent experiments. Asterisks represent significant differences from wild type among treatments (*p* < 0.05, *t* test).

Light-dependent autophosphorylation of phot1 and phot2 was readily detectable following dark acclimation (Figure 1A), as was reproducible arching of the photostimulated hypocotyl along the vertical agar surface (Figure 1B). Hence, dark acclimation can be used to restore phot activity to its ground state, thereby offering a means to study hypocotyl phototropism after photomorphogenesis.

After dark acclimation, phototropism within the first 12 h of treatment with low fluence rate blue light (1 µmol m^−2^ s^−1^) was attributed solely to the action of phot1 (Figure 1C), as observed in etiolated seedlings under equivalent light conditions [20]. Similarly, hypocotyls of *b19* mutants showed enhanced bending relative to wild type that was evident as early as 2–4 h after treatment (Figure 1C). Seedlings overexpressing *B19* displayed reduced initial rates of curvature (Figure 1C) consistent with the expanded auxin efflux activity in these transgenic lines [21]. In contrast to etiolated seedlings (Figure S1A), bending of dark-acclimated *pin3* hypocotyls was initially enhanced (Figure 1C), but was less than that of wild type when photostimulated beyond 12 h in both dark-grown and dark-acclimated seedlings (Figure S1B). However, bending in *pin3* mutants occurred at a higher position of the hypocotyl compared to wild type or *b19* mutants (Figure 1B), suggesting that PIN3 is spatially important for establishing phototropic curvature in dark-acclimated seedlings.

Prior to bending, hypocotyls of dark-acclimated seedlings showed reduced vertical growth in response to directional blue light, as reported for etiolated *Arabidopsis* seedlings [22] and rice coleoptiles [23], which was dependent on phot1 and not cry or phy photoreceptors (Figure 1D). Hypocotyl growth inhibition was still apparent in *pin3* mutants, whereas mutants lacking B19 showed comparable growth rates in dark and light but were reduced in comparison to *phot1* mutants (Figure 1D). Pooling of auxin in the upper hypocotyl region, including the cotyledonary node, through an interruption of B19 function rather than PIN3 would concur with the observed hypocotyl growth arrest that precedes phototropism.

### phot1 Activation Reduces Polar Auxin Transport

**Figure 2:**
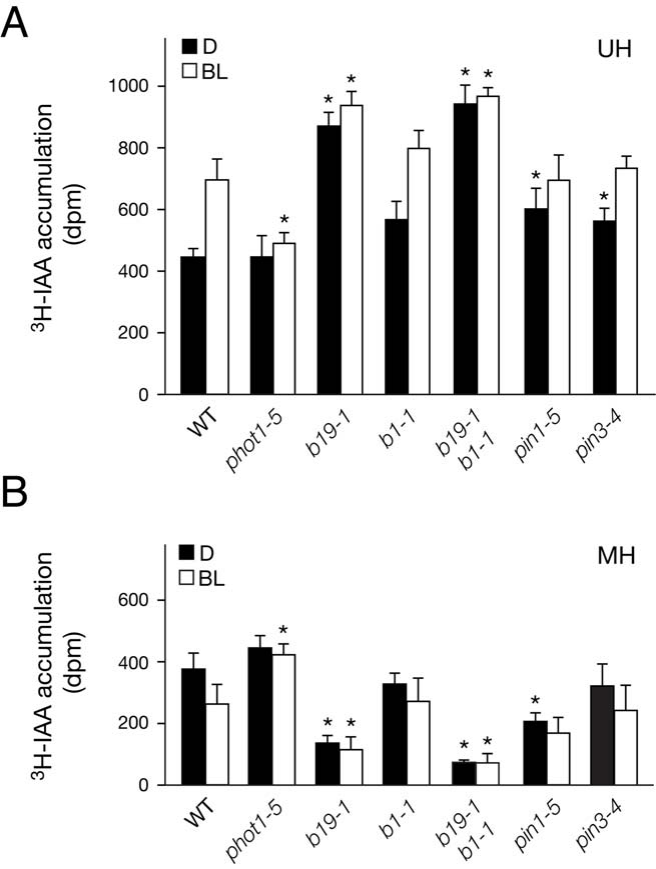
IAA transport in dark-acclimated seedlings. (A) ^3^H-IAA accumulation in the upper hypocotyl/cotyledonary node (UH). ^3^H-IAA (5 nl) was applied to ten seedling apices under a green safe light. Seedlings were exposed to directional blue light (BL, 1 µmol m^−2^ s^−1^) or continued darkness (D). Upper hypocotyls including the cotyledonary node were excised after 2.5 h. Mean hypocotyl lengths were 5 mm (wild type [WT] and *phot1–5*), 4.3 mm (*b19–1*), 4.8 mm (*b1–1*), 4.3 mm (*b1–1 b19–1*), 4.8 mm (*pin1–5*), and 4.5 mm (*pin3–4*). Results represent the mean + SD, *n  = *10 seedlings in three independent experiments. Asterisks represent significant differences from wild type among treatments (*p* < 0.05, *t* test). (B) ^3^H-IAA accumulation in the mid hypocotyl (MH) determined as in (A).

To determine whether the observed differences in vertical hypocotyl growth rates correlate with alterations in polar auxin flow, transport of the principle auxin indole-3-acetic acid (IAA) from the shoot apex was monitored in the presence or absence of directional blue light. Such an approach is not feasible with etiolated *Arabidopsis* seedlings since they lack a fully exposed shoot apex. ^3^H-IAA was applied to the shoot apex of dark-acclimated seedlings under safe light conditions. Seedlings were either retained in darkness or exposed to directional blue light (1 µmol m^−2^ s^−1^), and sections corresponding to the upper hypocotyl/cotyledonary node and the mid hypocotyl/elongation zone were harvested for radiotracer analysis. Measurements of ^3^H-IAA in these sections confirmed that directional blue light inhibits polar flow from the apical region (Figure 2A) to the mid hypocotyl (Figure 2B), resulting in IAA accumulation within the apical region. Moreover, blue-light-dependent inhibition of polar auxin transport was dependent on phot1, as *phot1* mutants lacked this response (Figure 2A and 2B).

Apical auxin retention by phot1 showed little dependence on PIN3, PIN1, and ABCB1 (abbreviated to B1), a second member of the ABCB transporter family, while a loss of B19 function increased retention (Figure 2A and 2B). Conversely, overexpression of *B19* reduced apical auxin (Figure S2). These findings suggest that B19 is a likely target of phot1 action to retain auxin within the upper hypocotyl within the first hour of blue-light exposure.

**Figure 3:**
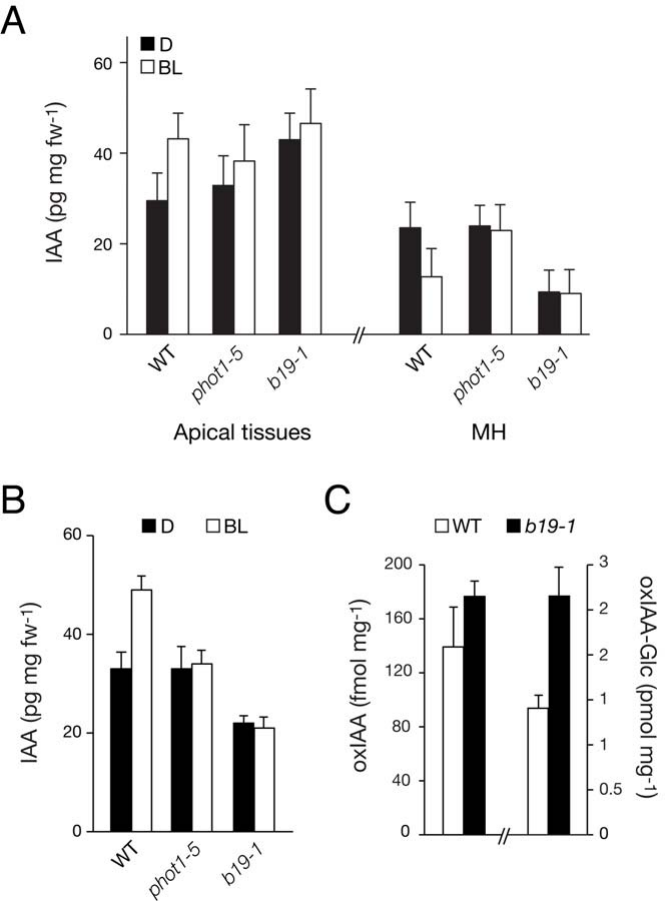
IAA accumulation in dark-acclimated seedlings. (A) Free IAA levels in wild-type (WT), *phot1–5,* and *b19–1* seedlings. IAA measurements were determined as described [43] after 2.5 h directional blue light (BL, 1 µmol m^−2^ s^−1^) or continued darkness (D). Apical tissues including the upper hypocotyl, petioles, and cotyledons were excised for analysis, in addition to the mid hypocotyl (MH). (B) Free IAA determinations as in (A), but after cotyledon excision. Apical tissues including the upper hypocotyl and cotyledonary node with the lower half of the petioles were excised for analysis. (C) Accumulation of auxin catabolites oxIAA and oxIAA-Glc in dark-acclimated wild-type and *b19–1* seedlings. Upper hypocotyls/cotyledonary nodes were excised and assayed. In each case, results represent the mean + SD, *n  = *100 seedlings in three independent experiments.

Quantification of endogenous IAA levels by gas chromatography–mass spectrometry showed a trend similar to that of the radiotracer studies (Figure 3A). phot1 mediated modest increases in IAA accumulation in the apical tissues of dark-acclimated seedlings that included the upper hypocotyl, petioles, and cotyledons (Figure 3A). However, endogenous IAA levels were actually lower in the apical tissues of *b19* seedlings after cotyledon removal (Figure 3B), whereas the levels of the IAA catabolism products oxindole-3-acetic acid (oxIAA) and oxIAA-glucose (oxIAA-Glc) were increased (Figure 3C). These findings suggest that sustained pooling of IAA in the shoot apex of *b19* mutants is sufficient to induce its catabolic activity [24]. The enhanced rates of phototropism typically associated with *b19* mutants most likely involve more dynamic or localized auxin accumulations than previously thought.

### phot1 Interacts with B19

**Figure 4:**
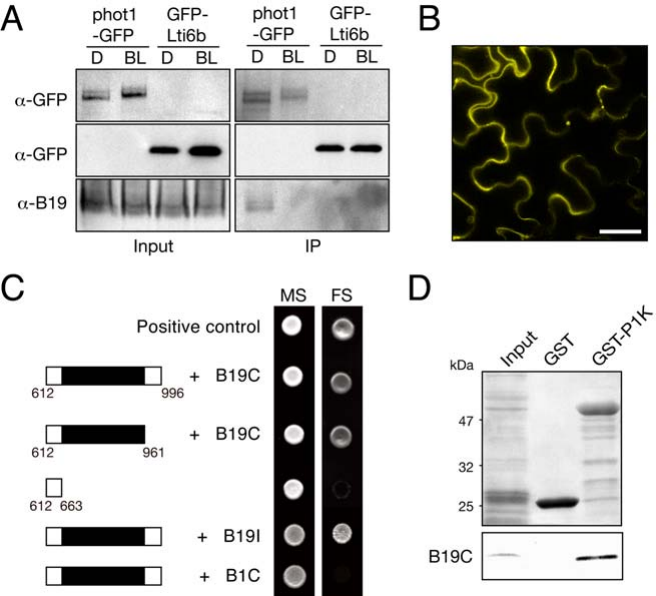
phot1 interacts with B19. (A) phot1-GFP and the plasma membrane marker fusion GFP-Lti6b were immunoprecipitated from membranes from 3-d-old etiolated seedlings (D) or seedlings exposed to blue light (BL, 20 µmol m^−2^ s^−1^) for 5 min. Samples were subjected to immunoblot analysis with anti-GFP and anti-B19 antibodies. Input (left) represents solubilized microsomes used for immunoprecipitation (IP, right). (B) BiFC fluorescence images of phot1-YN and B19-YC co-expressed in tobacco leaf epidermis. Reconstitution of YFP fluorescence was visible at the plasma membrane. Scale bar  = 20 µm. (C) The C-terminus of phot1 including its Ser/Thr kinase domain (amino acids 612–996) interacts with B19C (amino acids 1121–1252) in yeast. Growth on minimal selection medium (MS) selects for co-transformants, while growth on full selection medium (FS) selects for interacting proteins. Deletion of the catalytic subunit of phot1 kinase (shaded black, amino acids 663–961) results in a loss of interaction. phot1 kinase also interacts with the internal NBD of B19 (B19I, amino acids 480–660). No interaction was detected between phot1 kinase and the C-terminus of B1 (B1C, amino acids 1049–1286). Vectors encoding murine p53 and the SV40 large T-antigen were included as a positive control. (D) In vitro binding of B19C to phot1 kinase (P1K). Equivalent amounts of GST-P1K and GST (indicated in the upper panel by Ponceau S staining) were incubated with c-Myc-tagged B19C synthesized by in vitro transcription/translation (Input). Binding of B19C was visualized by immunoblot analysis with anti-c-Myc antibodies. Sizes of molecular weight markers are indicated on the left (kilodaltons).

To ascertain whether B19 represents a component of phot1 signaling, we performed co-immunoprecipitation using *Arabidopsis* expressing functional *PHOT1*:phot1–green fluorescent protein [GFP] [25]. B19 was found to co-immunoprecipitate specifically with phot1-GFP in darkness (Figure 4A). B19-phot1 interactions were also verified by mass spectrometry analysis of phot1-GFP and B19–hemagglutinin (HA) immunoprecipitates (Tables S1 and S2). In vivo irradiation prior to immnunoprecipitation of phot1-GFP attenuated its interaction with B19, implying that this interaction was transient upon phot1 kinase activation (Figure 4A), a property commonly associated with kinase-substrate interactions. A direct interaction between B19 and phot1 was further confirmed by bimolecular fluorescence complementation (BiFC). Reconstitution of a yellow fluorescent protein (YFP) signal at the plasma membrane was evident in tobacco epidermal cells co-expressing phot1 and B19 (Figure 4B), whereas no fluorescence was detected when either phot1 or B19 was expressed with empty vector controls (Figure S3).

The cytosolic C-terminus of B19 (B19C), encompassing its second nucleotide-binding domain (NBD), is important for its transporter activity and for mediating protein interactions [13],[26]. We found that B19C interacts with the C-terminus of phot1, including its Ser/Thr kinase domain, both in yeast (Figure 4C) and in in vitro co-immunoprecipitation assays (Figure 4D). Furthermore, domain mapping showed that the catalytic kinase subunit of phot1 was necessary for this interaction (Figure 4C). These data therefore suggest that B19 may function as a substrate for phot1 kinase activity. If this is the case, then substrate phosphorylation would be expected to be limited to B19 as no interaction was observed between phot1 kinase and the C-terminus of B1 in yeast (Figure 4C), despite its expression (Figure S4).

### phot1 Inhibits B19 Transporter Activity

**Figure 5:**
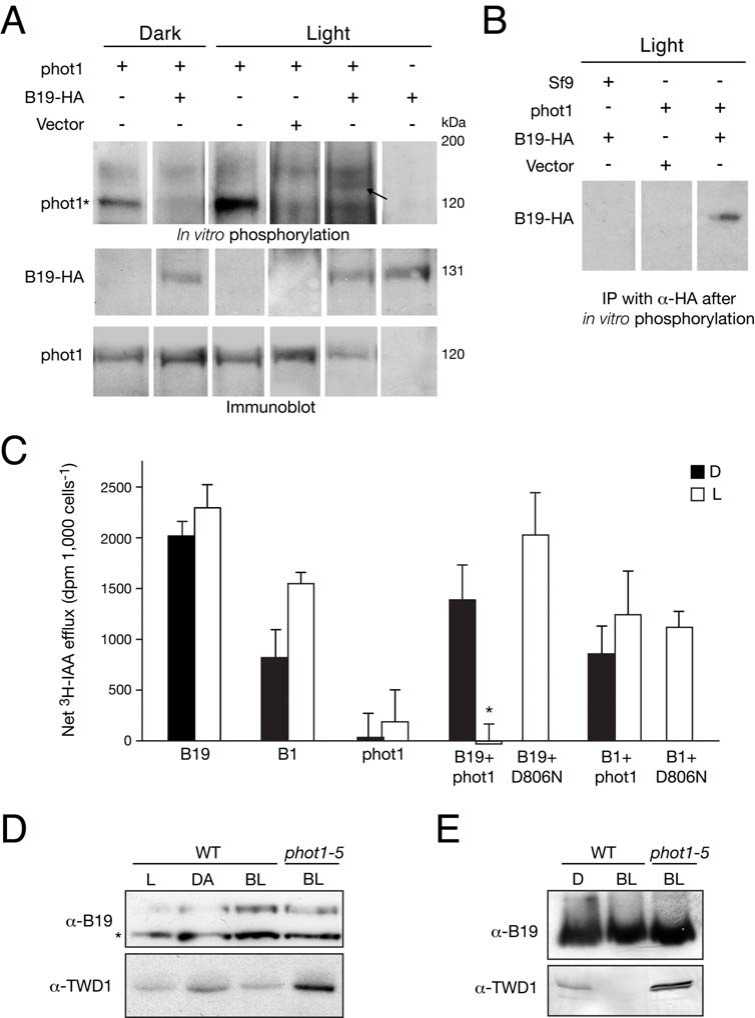
phot1 phosphorylation and regulation of B19 auxin transporter activity. (A) In vitro phosphorylation of B19 in the presence of phot1 (upper panel). Functional B19-HA was expressed in *S. pombe,* whereas active phot1 was expressed in *Spodoptera frugiperda* (Sf9) insect cells. Membranes containing B19-HA were mixed with phot1 enriched by nickel affinity chromatography. Samples were given a mock irradiation (D) or irradiated with 10,000 µmol m^−2^ white light (L) after the addition of radiolabeled ATP. The asterisk indicates phot1 autophosphorylation. B19-HA is phosphorylated in the presence of phot1 (indicated by the black arrow). *S. pombe* membranes expressing no B19-HA (vector only) were included as a control. B19-HA and phot1 protein levels were monitored using anti-HA and anti-phot1 antibodies, respectively (lower panels). Sizes of molecular weight markers are indicated on the right (kilodaltons). (B) Phosphorylation of B19-HA by phot1 in (A) was confirmed by immunoprecipitation (IP) of B19-HA using anti-HA antibodies (right lane). All samples were treated with light. No B19 phosphosignal was detected in the absence of B19 (center lane) or when Sf9 extracts lacking phot1 were used as a control (left lane). (C) phot1 regulation of B19-mediated auxin efflux in HeLa cells. Cells were prepared under a green safe light then incubated in darkness (D) or exposed to white light (L, 4 µmol m^−2^ s^−1^) for the duration of the assay. Light-treated samples alone are shown for both B19 and B1 co-expression with kinase inactive phot1 (D806N). Data are expressed as net ^3^H-IAA efflux (calculated from net ^3^H-IAA retention in cells expressing indicated proteins versus that of empty vector controls). Results represent mean + SD, *n  = *3. Three cell wells were used for each assay. The asterisk represents a significant difference between dark and light treatments (*p* < 0.05, *t* test). (D) To determine the effect of blue light on B19-TWD1 interactions in dark-acclimated seedlings, 3-d-old light-grown (L) *Arabidopsis* seedlings overexpressing TWD1 and functional B19-HA [12],[13] were subjected to dark acclimation (DA) prior to irradiation with directional blue light (BL, 2 µmol m^−2^ s^−1^) for 2 h. B19-HA was immunoprecipitated with anti-HA antibody and subjected to immunoblot analysis with anti-B19 and anti-TWD1 antibodies. No attenuation of B19-TWD1 interactions was observed when B19 was immunoprecipitated from the *phot1-5* mutant. The asterisk (*) indicates the position of B19. (E) Interaction analysis as in (D) but with 3-d-old dark-grown (D) seedlings.

Our attempts to express functional phot1 and B19 simultaneously in either insect cells or *Schizosaccharomyces pombe* were unsuccessful, precluding the possibility of investigating substrate phosphorylation by co-expression analysis in these systems. As an alternative, functional B19 generated in membranes of *S. pombe* was mixed with active phot1 enriched from insect cells for in vitro phosphorylation analysis. Although the addition of *S. pombe* membranes reduced the level of phot1 autophosphorylation from insect cells, increased phosphorylation of B19 in the presence of phot1 was apparent using this approach (Figure 5A). Phosphorylation of B19 in the presence of phot1 was verified by immunoprecipitation (Figure 5B), suggesting that B19 is a substrate target for phot1 kinase activity.

B19 is a stable plasma membrane protein that does not exhibit the dynamic relocalization characteristics of PIN transporters [12]. We therefore rationalized that B19 phosphorylation by phot1 could impact its transporter activity as opposed to its subcellular trafficking. To address this, we examined *Arabidopsis* ABCB transporter function in HeLa cells, as reported previously [13]. Expression of B19 and B1 resulted in auxin efflux in this system, whereas expression of phot1 was without effect (Figure 5C). In each case, light treatment of HeLa cells resulted in a modest increase in auxin efflux activity. However, co-expression with phot1 reduced B19 transporter activity, particularly following irradiation. Introduction of a single point mutation (D806N) shown previously to inhibit phot1 kinase activity [27] abrogated this effect, indicating that phot1 activation leads to a loss of B19 transporter function. Consistent with our yeast two-hybrid analysis (Figure 4C), phot1 had no impact on B1-mediated auxin transport.

While B19C interacts with phot1 kinase (Figure 4C and 4D), potential phosphorylation sites may include those residing within the first NBD of B19 [28], since this region was also found to interact with phot1 kinase (Figure 4C). One mechanism by which phot1 phosphorylation could impact B19 activity is by altering its interaction with key regulatory proteins. The immunophilin-type protein FKBP42/TWISTED DWARF 1 (TWD1) interacts directly with B19 [26] and is a positive regulator of B19-mediated auxin transport [21]. Co-immunoprecipitation of TWD1 with B19-HA was readily detected in dark-acclimated seedlings overexpressing TWD1 (Figure 5D). However, TWD1-B19 interactions were attenuated following 2 h of blue-light treatment. A similar effect of blue light was observed on TWD1-B19 interactions in etiolated seedlings (Figure 5E). In each case, a decrease in interaction between TWD1 and B19 following blue-light exposure was not observed in the *phot1* mutant, suggesting that phot1 could act to repress B19 activity in *Arabidopsis* by disrupting its interaction with TWD1.

### Lateral Auxin Fluxes Are Initiated above the Site of Phototropic Growth

Given the above results we conclude that localized pooling of auxin at and above the hypocotyl apex (Figure 2A), which coincides with reduced vertical growth at the onset of phototropism (Figure 1D), most likely results from a transient inactivation of B19 initiated by phot1 kinase activity. Consequently, auxin retention in the region immediately above the hypocotyl apex and not the elongation zone would serve to prime subsequent lateral fluxes required for phototropism [7],[23]. In monocots, lateral auxin redistribution is proposed to occur at the coleoptile tip, the predominant site of asymmetric phot1 activation [29]. Our results therefore suggested that a similar mechanism of phototropic detection exists in *Arabidopsis*. To address this, we monitored cellular auxin accumulation using the synthetic transcriptional reporter *DR5rev*:GFP. Activity of the auxin-responsive promoter *DR5* has been used to visualize the spatial pattern of auxin responses, and hence, indirectly, the distribution of auxin [30].

**Figure 6:**
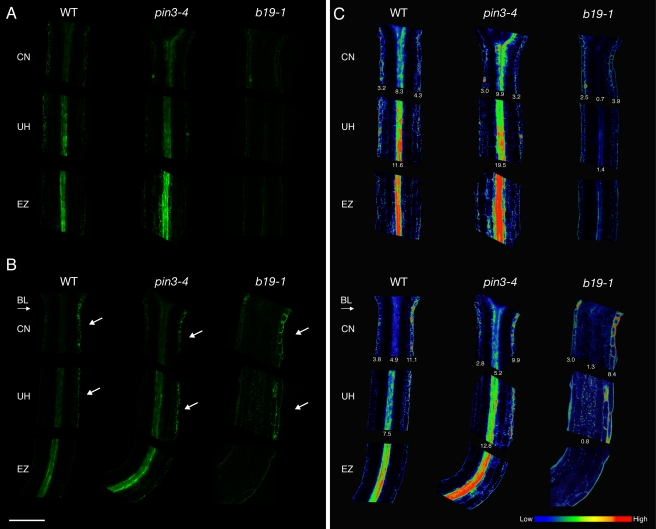
Blue-light-driven lateral auxin gradients in dark-acclimated hypocotyls. (A) Auxin distributions in dark-acclimated wild-type (WT), *pin3–4,* and *b19–1* seedlings were monitored using the auxin reporter *DR5rev*:GFP. *DR5rev*:GFP signals in the cotyledonary node (CN), upper hypocotyl (UH), and elongation zone (EZ). Note the lack of signal in the central vasculature in *b19–1*. (B) *DR5rev*:GFP signals in hypocotyls exposed to a directional blue light (BL, 1 µmol m^−2^ s^−1^) for 3 h. In all genotypes, signals are asymmetrically localized in the epidermal cells on the shaded side of the hypocotyl (white arrows) in both the CN and UH. (C) Heat map representation of *DR5rev*:GFP signals shown in (A) and (B). Quantification of the mean pixel intensity of the *DR5rev*:GFP signals associated with the epidermal regions of the CN are shown, as are the central vasculature signals for the CN and UH. Quantification was performed using the analyze and measure functions within ImageJ (version 1.43u). Data are representative of *n = *20 seedlings in three independent experiments for wild type and *n = *10 in two separate experiments for *pin3–4* and *b19–1*. Scale bar = 200 µm.

In wild type, *DR5rev*:GFP was readily detected in the epidermis and central vasculature of the cotyledonary node and upper hypocotyl (Figure 6A). Below in the elongation zone, *DR5rev*:GFP was associated predominantly with the central vasculature. Phototropic stimulation produced an asymmetric signal in the epidermis encompassing the cotyledonary node and upper hypocotyl after 3 h (Figure 6B) that was apparently phot dependent (Figure S5A).

Heat map representation and quantification of the *DR5rev*:GFP fluorescence imaging confirmed that there was an in increase in signal on the shaded versus the irradiated side of the hypocotyl apex and cotyledonary node (Figure 6C). Consistent with phot1 inhibition of B19-mediated auxin loading into, and retention within, provascular/vascular tissues, *DR5rev*:GFP signals decreased simultaneously in the vasculature of the elongation zone of wild-type hypocotyls (Figure 6B and 6C). However, no *DR5rev*:GFP asymmetry was detected in this epidermal region despite the onset of curvature. Thus, the primary site of asymmetric auxin accumulation following light perception by phot1 occurs at and above the hypocotyl apex, not in the region of differential growth.

### Lateral Auxin Fluxes Occur in *b19* and *pin3* Mutants

Lateral *DR5rev*:GFP signals were still apparent in *b19* and *pin3* seedlings following phototropic stimulation (Figure 6B and 6C). Asymmetric auxin gradients were transient and no longer detectable following a 12-h period of irradiation, except in *b19* mutants, where a weak asymmetric signal persisted in the epidermis of the shaded side of the hypocotyl (Figure S5B). More rapid diversion of auxin out of vascular tissues in and above the hypocotyl apex of *b19* mutants (Figure 2A) may account for this sustained lateral signal.

**Figure 7:**
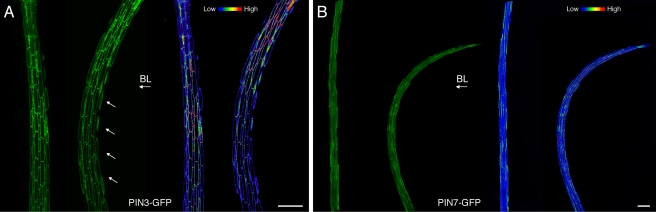
PIN3- and PIN7-GFP localization in dark-acclimated seedlings. (A) Functional *PIN3*:PIN3-GFP fluorescence in hypocotyls of seedlings exposed to blue light (BL, 1 µmol m^−2^ s^−1^) for 6 h or continued darkness. A blue-light-treated seedling is shown on the right and white arrows indicate a decrease in *PIN3*:PIN3-GFP signal. Scale bar  = 200 µm. (B) Functional *PIN7*:PIN7-GFP fluorescence in hypocotyls of seedlings exposed to blue light (1 µmol m^−2^ s^−1^) for 12 h or continued darkness. Scale bar  = 20 µm. Heat map representations of GFP signals are shown on the right. In each case data are representative of *n*>20 seedlings.

In comparison to wild type, *DR5rev*:GFP signals were reduced in the central vascular tissue of *b19* hypocotyls (Figure 6A), consistent with both IAA, oxIAA, and oxIAA-Glc determinations (Figure 3) and the cellular localization of *B19*:B19-GFP (Figure S6). Dynamic pooling and catabolism of IAA within the central vasculature of *b19* seedlings may contribute to a cellular auxin status that is too low for *DR5rev*:*GFP* to report. However, we cannot exclude the possibility that sustained IAA overaccumulation in *b19* mutants could have an adverse effect on *DR5rev*:GFP reporter activity. In contrast to *b19* mutants, a strong *DR5rev*:GFP signal was observed in the central vasculature of *pin3* mutants (Figure 6A). Moreover, the *DR5rev*:GFP fluorescence appeared to be more diffuse throughout the bundle sheath, consistent with the role of PIN3 in redirecting auxin back into the central cylinder [31]. Our radiotracer studies also suggested that PIN3, like PIN1, participates in auxin transport out of the hypocotyl apex and the region immediately above (Figure 2A). In addition, functional *PIN3*:PIN3-GFP fluorescence levels became reduced in and below the region of elongation in dark-acclimated seedlings following phototropic stimulation for 6 h (Figure 7A). By comparison, directional blue light had no effect on the level of *PIN7*:PIN7-GFP fluorescence even after 12 h of blue-light exposure (Figure 7B). The reduced abundance of PIN3 in the lower hypocotyl following phototropic stimulation, combined with the higher position of curvature associated with *pin3* (Figure 1B) and *pin3 b19* mutants (Table 1), led us to propose that PIN3 is involved in channeling auxin to the region of differential growth once lateral redistribution has been established in and immediately above the hypocotyl apex.

**Table 1:** Phototropic bending in auxin transport mutants of *Arabidopsis*.

Genotype	Mean Bending Angle (8 h) ± SD	Student’s *t* Test Compared to Col-0	Notes
Col-0	26±2.4	ND	
*pin1–1*	20±6.8	ND	
*pin1–5*	23±3.6	ND	
*pin2 (agr1–1, eir1–1)*	27±2.3	ND	
*pin3–4*	33±1.6	>*p* = 0.014	Bends at a higher position in the hypocotyl
*pin4–1*	25±2.8	ND	
*pin7* (N548791)	18±3.0	< *p* = 0.023	
*pin1–5 pin3–4*	28±4.7	ND	
*pin3–4 b19–1*	34±2.9	>*p* = 0.021	Bends at a higher position in the hypocotyl
*b1–1*	28±3.5	ND	
*b4–1*	27±3.6	ND	
*b19–1*	39±2.2	>*p* = 0. 023	
*b1–1 b4–1 b19–1*	40±3.8	>*p* = 0.006	
*pin1–1 b1–1 b19–1*	34±6.6	ND	
*aux1–7*	23±4.5	ND	
*aux1 lax2 lax3*	21±3.3	*p* = 0.101	

Homozygous dark-acclimated seedlings were analyzed for phototropic bending induced by 8 h directional blue light (1 µmol m^−2^ s^−1^). Results represent the mean ± SD, *n = *10 seedlings in three independent experiments except for *pin1–1* and *pin1–1 b1–1 b19–1,* where eight and five seedlings were used, respectively, in each experiment. For assays of *pin1–1* and *pin1–1 b1–1 b19–1*, progeny of a single plant heterozygous for *pin1–1* were used in the bending assays. Reduced-bending *pin1–1* seedlings and increased-bending *pin1–1 b1–1 b19–1* seedlings were selected and grown to maturity then examined for pin-formed inflorescences to determine if they were homozygous for *pin1*. Significant differences compared to wild type (Col-0) are indicated. ND, not determined.

## Discussion

### *Arabidopsis* Sheds New Light on the Cholodny-Went Theory

**Figure 8:**
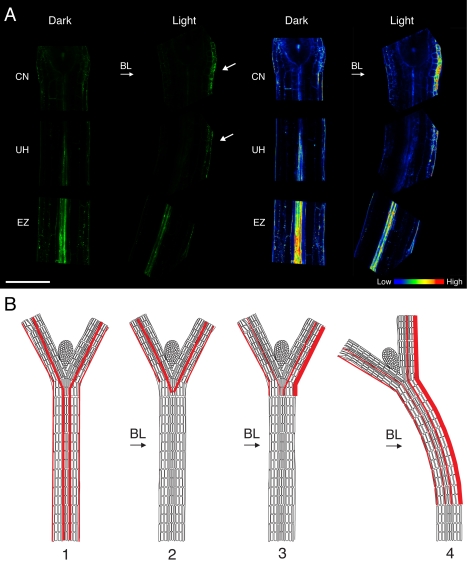
Auxin movements associated with phototropism in dark-acclimated *Arabidopsis*. (A) Auxin distributions in dark-acclimated wild-type seedlings where the cotyledons were excised prior to analysis. *DR5rev*:GFP signals in dark- or light-treated seedlings were monitored as in Figure 6. Heat map representation of *DR5rev*:GFP signals is shown on the right. BL, blue light; CN, cotyledonary node; EZ, elongation zone; UH, upper hypocotyl. (B) Schematic model of auxin movements associated with phototropism. Red lines represent polar auxin movement from the shoot to the root. Weight of the lines represents the relative amount of auxin movement. (1) In darkness, auxin primarily moves through the vascular tissues in the petioles and hypocotyl, and also through epidermal tissues, (2) Upon exposure to directional blue light, auxin movement is temporarily halted at the cotyledonary node and the seedling stops elongating vertically. (3) This is followed by a redistribution of auxin at the cotyledonary node to the shaded side of the seedling and a channeling to the elongation zone below. (4) Subsequent elongation of the cells on the shaded side of the hypocotyl results in differential growth and bending towards the light source.

In recent years, *Arabidopsis* has been used extensively as a genetic model to dissect the light-sensing and signaling mechanisms associated with phototropism [1]. However, research to date has focused on etiolated seedlings that simultaneously undergo photomorphogenesis in addition to phototropism when irradiated with directional blue light. In the present study, we show that it is possible to probe auxin fluxes associated with phototropism after such de-etiolation events are fully established. Dark-acclimated light-grown seedlings, like etiolated seedlings [20], are phototropically responsive (Figure 1A and 1B), but provide a more amenable system to study auxin transport processes in response to blue-light irradiation (Figure 2A and 2B). The open cotyledons of light-grown seedlings do influence the curvature formation of photostimulated hypocotyls owing to their friction against the vertical agar surface, which must be uniformly maintained by experimental conditions. Nevertheless, the contribution of the cotyledons to the curvature response itself is likely to be minimal, as both phototropism and the establishment of lateral auxin fluxes can be detected in seedlings where the cotyledons have been removed prior to photostimulation (Figure 8A).

With regard to phototropism, we draw two main conclusions from this work. First, the lateral auxin translocation system in dark-acclimated *Arabidopsis* seedlings complies with the Cholodny-Went theory of tropic curvature proposed for monocots in the late 1920s [32]. This hypothesis predicts that asymmetric light is perceived at the coleoptile tip and causes auxin to move from the irradiated to the shaded side. Auxin then moves down the coleoptile, where the higher concentration on the shaded side promotes differential growth towards light as a result of increased cell elongation. Although subject to many criticisms since it was first introduced, the Cholodny-Went theory has received considerable support from monocot studies [1],[3]. However, the apex has generally not been regarded as the site of phototropic perception in dicots [33]. Rather, studies of the auxin transport processes associated with *Arabidopsis* phototropism have focused on possible redistribution of auxin out of the central vasculature at the hypocotyl elongation zone [6].

Here we demonstrate that the Cholodny-Went theory holds true for dicot phototropism, where the primary site of lateral auxin translocation resides at and above the hypocotyl apex (Figure 6). Quantification of *DR5rev*:GFP signals within the upper hypocotyl in wild type and *pin3* mutants suggests that the increase in auxin in the epidermis of the shaded side of the hypocotyl coincides with a decrease in signal from the central vasculature (Figure 6C). This differs from the situation in monocots, where a net movement from the irradiated to the shaded side of the coleoptile is thought to establish the auxin asymmetry [23]. However, it is worth noting that this conclusion is drawn from radiotracer studies that monitor the effect of light on polar IAA transport within two separate halves of the coleoptile [1],[3]. A spatial assessment of cellular auxin levels will be necessary to determine whether lateral auxin translocation in monocots is established differently from what is observed in dark-acclimated *Arabidopsis*.

### A Model for Dicot Phototropism

The results presented here also provide new mechanistic information about the development of hypocotyl phototropism in *Arabidopsis* (proposed schematically in Figure 8B). In particular, our studies uncover a direct connection between the primary photoreceptor for phototropism, phot1, and the auxin transporter B19. More specifically, B19 functions as a target for phot1 action in the shoot apical tissues to initially halt vertical growth (Figure 1D) and concentrate auxin within this region (Figure 2A and 2B). These findings are consistent with earlier reports showing that an inhibition of polar auxin transport [34], as well as light-mediated growth inhibition [22],[23],[35], precedes the onset of phototropic curvature.

B19 is directly regulated by phot1 kinase activity, identifying it as a likely substrate for this class of photoreceptor kinase (Figures 4 and 5A–5C). Regulation of B19 function *in planta* may require more complex phosphorylation events involving other kinases. Reconstitution of active B19 and phot1 in artificial membranes would aid analyses of their interactions, although such efforts have not been successful to date. Our data also suggest that in vivo interactions between phot1 and B19 are likely to be transient following irradiation (Figure 4A). This finding is not unexpected given that phot1 is rapidly internalized from the plasma membrane following blue-light excitation [25] and receptor autophosphorylation [36]. A transient interaction would also ensure that B19 transporter activity is not continually inhibited by phot1 action. One possible consequence of phot1 phosphorylation could be to disrupt an interaction between B19 and its positive regulator TWD1 (Figure 5D and 5E). If so, the FKBP38 mammalian ortholog of TWD1 [21] would also serve as a target of phot1 action on B19 transporter activity in HeLa cells (Figure 5C). Studies are now underway to test this potential mode of regulation (Figure S7).

Following its transient pooling in apical perivascular tissues, auxin is subsequently translocated from the central vasculature to the epidermis at the shaded side, where PIN3 participates in its transport to the elongation zone below. Retention of auxin in the elongation zone is facilitated by a reduction in PIN3 abundance in and below this region of the hypocotyl after phototropic stimulation (Figure 7A). Without PIN3, auxin is unable to move downward to this region. Consequently, localized apical pooling results in faster initial rates of curvature (Figure 1C) at a higher region of the hypocotyl (Figure 1B). This is not apparent with etiolated *pin3* seedlings, where curvature is reduced (Figure S1A), an outcome possibly arising from PIN3’s impact on apical hook opening [9] as reported for PIN1 [13].

### Lateral Auxin Translocation Processes

The second conclusion to be drawn from this work is that the lateral translocation of auxin required for phototropism does not depend on B19 or PIN3 (Figures 1C and 6). Indeed, this process cannot be ascribed to any of the well-characterized auxin transporters, as mutants lacking these proteins are phototropic in our dark-acclimated system despite showing some altered rates in bending (Table 1). In light of these results, efforts to contrive a response mechanism involving these components are not convincing. Coordinated action between multiple transporters is therefore likely to drive the lateral redirection of auxin that is necessary to establish phototropic growth. PIN7 could be a factor in the lateral redistribution process, in conjunction with other transporters, as dark-acclimated *pin7* seedlings are phototropic, but exhibit lower curvatures relative to wild type (Table 1).

The mechanism by which lateral auxin gradients are established is still unknown. As demonstrated here, phosphorylation of the transporters involved by phototropic receptors offers one possible mode of regulation. phot1 could control the subcellular trafficking of regulatory components of the lateral relocation process, as recent studies have identified ADP-ribosylation factors as direct interaction targets [37]. Lateral translocation may also be directed by differential proton extrusion, which represents a primary driving force for both auxin uptake and efflux [4]. Further studies of NON-PHOTOTROPIC HYPOCOTYL 3 (NPH3) will undoubtedly be important to improve our understanding of the processes involved, as mutants lacking this protein are aphototropic [38] and fail to exhibit lateral auxin transport [23]. NPH3 is a plant-specific plasma-membrane-associated protein that directly interacts with phot1 [38]. Photoactivation of phot1 leads to dephosphorylation of NPH3, a signaling process that has been linked to the onset of phototropic curvature [39], but how NPH3 functions to orchestrate auxin transport regulation remains poorly understood. Members of the PHYTOCHROME KINASE SUBSTRATE (PKS) protein family are known to play a role in the establishment of phototropic curvatures [40], in addition to other phot-mediated responses [41]. We anticipate that adoption of this revised system to study phototropism will accelerate our understanding of the role of these, and as yet undiscovered, components of phototropic signaling.

## Materials and Methods

### Plant Material and Growth

The *Arabidopsis thaliana* ecotype Col-0 was used throughout this study. Details of each line can be found in Table S3. Unless otherwise stated, seedlings were grown on 0.8% agar plates containing ¼ Murashige Skoog basal salts (pH 5.5) supplemented with 0.5% sucrose for 3 d under white light (100 µmol m^−2^ s^−1^). During growth, plates were tilted 2° back from the vertical. Plates were transferred to darkness in light-tight gas exchange boxes equipped with directional 450-nm blue-light LED arrays (Roithner Lasertechnik) calibrated to 1 µmol m^−2^ s^−1^ and an infrared light source (Electro Optical Components) and tilted 1° forward for 24 h to dark acclimate. Subsequent growth in light or darkness was measured using a CCD video camera (Sony) equipped with a narrow band pass infrared filter (Electro Optical Components). Images were quantified using Image J software (US National Institutes of Health). Phototropism in etiolated seedlings was performed as described [42].

### Auxin Transport and Quantification

Auxin transport and quantification was performed as described [13],[26]. ^3^H-IAA at 10 µM (30 Ci mmol^−1^, American Radiolabeled Chemicals) was dissolved in ethanol. ^3^H-IAA was adsorbed to the surface of 125-µm to 212-µm polystyrene beads (Sigma) and placed at the hypocotyl apex by touching the tips of the true leaves with a micromanipulator and liquid stream from a pipettor. All experiments were prepared under green or red safe lights. Seedlings were incubated in darkness or directional blue light (1 µmol m^−2^ s^−1^) for the times indicated. Sections were collected under white light, with cuts through the true leaves/cotyledonary petioles to remove sites of contact with polystyrene beads and hypocotyl immediately below the cotyledonary node. A second section, corresponding to the mid hypocotyl and elongation zone, was collected by making a second cut in the hypocotyl 2 mm below. Free IAA determination was performed as described [43] on sections made from the mid hypocotyl and apical tissues including the upper hypocotyls, petioles, and cotyledons. oxIAA and oxIAA-Glc levels were assayed by liquid chromatography–mass spectrometry using a Waters Micromass Q-TOF micro and Agilent HPLC-MSD/TOF system calibrated to a standard curve generated with a defined mixture of IAA, oxIAA, and oxIAA-Glc using ^13^C-IAA (Cambridge Isotopes) as an internal standard.

### Auxin Efflux Assays in HeLa Cells

Assays were performed as described [13],[44] in darkness or under a green safe light unless otherwise stated. Cells were transfected with pTM1 vectors encoding *B19* and *PHOT1* in a ratio of 4∶1, respectively. Co-expression of *PHOT1* and *B19* was verified by real time quantitative PCR and immunolocalization as described [13].

### Immunoprecipitation from *Arabidopsis*

Membrane proteins were extracted and GFP immunoprecipitation performed as described [42]. Proteins were identified by liquid chromatography–tandem mass spectrometry using the Fingerprints Proteomics Facility (University of Dundee). B19 immunoprecipitation was performed as described [12],[13], and proteins identified by using liquid chromatography–matrix-assisted laser desorption/ionization mass spectrometry analysis (Bindley Bioscience Center, Purdue University).

### Yeast Two-Hybrid Analysis

Interaction assays were carried out as described [37]. Interactions between regions of *B19* cDNA and *PHOT1* cDNA were performed using pGADT7 prey and pGBKT7 bait vectors, respectively (Clontech). The C-terminal region of B1 used for yeast two-hybrid analysis was as described [26].

### In Vitro Co-Immunoprecipitation

Expression of GST-fusion proteins and GST was performed as described [37]. The TNT System (Promega) was used to generate c-Myc-tagged protein from 2-µg circular plasmid DNA (pGBKT7). The MagneGST Protein Purification System (Promega) was used for in vitro GST pull-down assays in accordance to the manufacturer’s instructions. Proteins were eluted in 1× SDS-PAGE loading buffer and subjected to SDS-PAGE and immunoblotting with anti-c-Myc antibodies (Roche).

### In Vitro Kinase Assays

B19-HA was expressed in *S. pombe* [45]. Sf9 insect cells were used to express phot1 harboring a 6xHis epitope [27]. Sf9 extracts were passed through a HisSpinTrap column (GE Healthcare) in accordance with the vendor’s protocol. Eluted phot1 was desalted using a microcon 1000 centrifugal filter (Millipore), and phosphorylation assays performed [27] in 20-µl reactions with 3 µg of phot1 extract and 10 µg of *S. pombe* membranes containing B19-HA. Immunoprecipitation of B19-HA following in vitro phosphorylation assays was performed as described [46] using anti-HA antibodies (Santa Cruz Biotechnology) and Immunobead Reagent (Bio-Rad). Kinase assays with plant membranes were performed as reported [42].

### Confocal Microscopy

Images were collected using a Zeiss LSM 710 confocal laser-scanning microscope with a 40× water immersion objective (1.4 numerical aperture, C-Apochromatic) and argon laser. Excitation was fixed at 488 nm using the primary dichroic mirror, and the Meta detector was used to capture GFP (492–533 nm) emission. Attenuation of the 25-mW argon laser was set at 30%. Master gain was set to 812, and the digital offset to 4. Pinhole size was 78 µm. No autofluorescence was observed in wild-type seedlings using these settings. Bright-field images were acquired simultaneously using the transmission detector. For BiFC, the coding sequence of *B19* was cloned via BamHI/SmaI into the binary vector pSPYCE-35S, and assays performed as described [36].

## Supporting Information

Figure S1 **Phototropic responsiveness of** ***pin3*** **mutants.** (A) Phototropic response of 3-d-old etiolated wild-type (WT) and *pin3-4* seedlings. Directional blue light (1 µmol m^−2^ s^−1^) was supplied for 12 h. Results represent the mean ± standard error, *n = *10 seedlings. (B) Phototropic response of dark-acclimated wild-type and *pin3-4* seedlings. Seedlings were subjected to directional blue light (1 µmol m^−2^ s^−1^) for the times indicated. Results represent the mean + standard error, *n = *10 seedlings.
(TIF)

Figure S2 **^3^H-IAA accumulation in dark-acclimated seedlings overexpressing** ***B19*** **(** ***b19oe*** **).** Seedlings were exposed to directional blue light (BL) (1 µmol m^−2^ s^−1^) or continued darkness (D). Upper hypocotyls including the cotyledonary node (UH) and mid hypocotyls including the elongation zone (MH) were excised after 2.5 h. Results represent the mean + SD, *n = *10 seedlings in three independent experiments.
(TIF)

Figure S3 **Bright-field BiFC fluorescence images of phot1-YN and B19-YC co-expressed in tobacco epidermal cells.** Reconstitution of YFP fluorescence between phot1-YN and B19-YC was visible at the plasma membrane (left). Only background YFP signals were detected for phot1-YN and YC (center) or YN and B19-YC (right). Scale bar  = 20 µm and applies to all images.
(TIF)

Figure S4 **Expression of phot1 kinase and the C-terminal NBD of B1 in yeast.** Immunoblot analysis of yeast co-expressing phot1 kinase (P1K) and the C-terminal NBD of B1 (B1C). Protein extracts (10 µg) co-expressing phot1 kinase and either B1C (1) or empty vector controls (2) were probed with anti-GAL4 DNA-binding domain and activation domain antibodies to discriminate phot1 kinase and C-terminal B1 proteins, respectively.
(TIF)

Figure S5 **Auxin accumulation in the upper hypocotyl/cotyledonary node of dark-acclimated hypocotyls.** (A) *DR5rev*:GFP signals in *phot1–5 phot2–1* seedlings exposed to directional blue light (BL, 1 µmol m^−2^ s^−1^) for 3 h. Signal intensity of *DR5rev*:GFP was noticeably lower in comparison to the other lines examined, giving rise to higher background plastid autofluorescence (represented by spots). (B) *DR5rev*:GFP signals in seedlings exposed to directional blue light for 12 h. Note the lack of signal in the vascular bundle in *b19–1* seedlings and the reduced signal in the vascular bundle below the upper hypocotyl in *pin3–4* seedlings. Data are representative of *n*>5. In each case, scale bar  = 200 µm.
(TIF)

Figure S6 **B19-GFP localization in dark-acclimated seedlings.** Functional *B19*:B19-GFP fluorescence is restricted to the central vasculature and epidermis (white arrows). Central spots represent plastid autofluorescence. Data are representative of *n*>20. Scale bar  = 100 µm.
(TIF)

Figure S7 **Model depicting a potential mechanism for the blue-light-dependent inhibition of B19-mediated auxin transport activity.** In darkness or ground state conditions, B19 (blue, center) actively exports IAA from the cytosol via interactions with its positive regulator TWD1. Under these conditions, B19 also interacts with the blue-light photoreceptor phot1. In response to blue-light exposure, phot1 undergoes autophosphorylation and transphosphorylates B19. Sites of B19 phosphorylation may also be targets for other regulatory kinases. Phosphorylation of B19 may promote an alteration in protein structure that disrupts its interaction with TWD1, leading to an inhibition of IAA efflux. phot1 is internalized upon receptor autophosphorylation, creating an inhibitory mechanism that is transient and becomes reactivated upon dephosphorylation of B19 and phot1 by as yet unidentified protein phosphatases.
(TIF)

Table S1 **Liquid chromatography–tandem mass spectrometry analysis of phot1-GFP immunoprecipitates.** Proteins identified with a Mascot score >100 are shown. *^a^PHOT1*:phot1-GFP was immunoprecipitated from 3-d-old etiolated *Arabidopsis* seedlings kept in darkness (D) or exposed to a blue light (BL, 20 µmol m^−2^ s^−1^) for 5 min. ^b^TAIR AGI numbers given where mass spectrometry data match a single accession; “various” denotes multiple isoforms were identified.
(DOC)

Table S2 **Liquid chromatography–matrix-assisted laser desorption/ionization mass spectrometry analysis of B19 immunoprecipitates.** Proteins identified with a Mascot score >150 are shown. TAIR AGI numbers are given. *B19*:B19-HA was immunoprecipitated from 5-d-old *Arabidopsis* seedlings. ^a^Previously identified in B19 fractions [12].
(DOC)

Table S3 **Lines used in this study.**
(DOC)

## Abbreviations

B19C: C-terminus of B19
BiFC: bimolecular fluorescence complementation
GFP: green fluorescent protein
IAA: indole-3-acetic acid
HA: hemagglutinin
NBD: nucleotide-binding domain
oxIAA: oxindole-3-acetic acid
SD: standard deviation
YFP: yellow fluorescent protein

## References

[1] Holland J. J, Roberts D, Liscum E (2009). Understanding phototropism: from Darwin to today.. J Exp Bot.

[2] Darwin C (1880). The power of movement in plants..

[3] Whippo C. W, Hangarter R. P (2006). Phototropism: bending towards enlightenment.. Plant Cell.

[4] Vanneste S, Friml J (2009). Auxin: a trigger for change in plant development.. Cell.

[5] Christie J. M (2007). Phototropin blue-light receptors.. Annu Rev Plant Biol.

[6] Friml J, Wiśniewska J, Benková E, Mendgen K, Palme K (2002). Lateral relocation of auxin efflux regulator PIN3 mediates tropism in *Arabidopsis*.. Nature.

[7] Esmon C. A, Tinsley A. G, Ljung K, Sandberg G, Hearne L. B (2006). A gradient of auxin and auxin-dependent transcription precedes tropic growth responses.. Proc Natl Acad Sci U S A.

[8] Křeček P, Skůpa P, Libus J, Naramoto S, Tejos R (2009). The PIN-FORMED (PIN) protein family of auxin transporters.. Genome Biol.

[9] Zádníková P. J, Petrásek J, Marhavy P, Raz V, Vandenbussche F (2010). Role of PIN-mediated auxin efflux in apical hook development of *Arabidopsis thaliana*.. Development.

[10] Nagashima A, Uehara Y, Sakai T (2008). The ABC subfamily B auxin transporter AtABCB19 is involved in the inhibitory effects of N-1-naphthyphthalamic acid on the phototropic and gravitropic responses of *Arabidopsis* hypocotyls.. Plant Cell Physiol.

[11] Mravec J, Kubes M, Bielach A, Gaykova V, Petrásek J (2008). Interaction of PIN and PGP transport mechanisms in auxin distribution-dependent development.. Development.

[12] Titapiwatanakun B, Blakeslee J. J, Bandyopadhyay A, Yang H, Mravec J (2009). ABCB19/PGP19 stabilizes PIN1 in membrane microdomains in *Arabidopsis*.. Plant J.

[13] Blakeslee J. J, Bandyopadhyay A, Lee O. R, Mravec J, Titapiwatanakun B (2007). Interactions among PIN-FORMED and P-glycoprotein auxin transporters in *Arabidopsis*.. Plant Cell.

[14] Wu G, Cameron J. N, Ljung K, Spalding E. P (2010). A role for ABCB19-mediated polar auxin transport in seedling photomorphogenesis mediated by cryptochrome 1 and phytochrome B.. Plant J.

[15] Noh B, Bandyopadhyay A, Peer W. A, Spalding E. P, Murphy A. S (2003). Enhanced gravi- and phototropism in plant *mdr* mutants mislocalizing the auxin efflux protein PIN1.. Nature.

[16] Nagashima A, Suzuki G, Uehara Y, Saji K, Furukawa T (2008). Phytochromes and cryptochromes regulate the differential growth of *Arabidopsis* hypocotyls in both a PGP19-dependent and a PGP19-independent manner.. Plant J.

[17] Tsuchida-Mayama T, Sakai T, Hanada A, Uehara Y, Asami T (2010). Role of the phytochrome and cryptochrome signaling pathways in hypocotyl phototropism.. Plant J.

[18] Parks B. M, Quail P. H, Hangarter R. P (1996). Phytochrome A regulates red-light induction of phototropic enhancement in *Arabidopsis*.. Plant Physiol.

[19] Whippo C. W, Hangarter R. P (2003). Second positive phototropism results from coordinated co-action of the phototropins and cryptochromes.. Plant Physiol.

[20] Sakai T, Kagawa T, Kasahara M, Swartz T. E, Christie J. M (2001). *Arabidopsis* nph1 and npl1: blue light receptors that mediate both phototropism and chloroplast relocation.. Proc Natl Acad Sci U S A.

[21] Bouchard R, Bailly A, Blakeslee J. J, Oehring S. C, Vincenzetti V (2006). Immunophilin-like TWISTED DWARF1 modulates auxin efflux activities of *Arabidopsis* P-glycoproteins.. J Biol Chem.

[22] Folta K. M, Spalding E. P (2001). Unexpected roles for cryptochrome 2 and phototropin revealed by high-resolution analysis of blue light-mediated hypocotyl growth inhibition.. Plant J.

[23] Haga K, Takano M, Neumann R, Iino M (2005). The Rice COLEOPTILE PHOTOTROPISM1 gene encoding an ortholog of *Arabidopsis* NPH3 is required for phototropism of coleoptiles and lateral translocation of auxin.. Plant Cell.

[24] Östin A, Kowalyczk M, Bhalerao R. P, Sandberg G (1998). Metabolism of indole-3-acetic acid in *Arabidopsis*.. Plant Physiol.

[25] Sakamoto K, Briggs W. R (2002). Cellular and subcellular localization of phototropin 1.. Plant Cell.

[26] Geisler M, Kolukisaoglu H. U, Bouchard R, Billion K, Berger J (2003). TWISTED DWARF1, a unique plasma membrane-anchored immunophilin-like protein, interacts with *Arabidopsis* multidrug resistance-like transporters AtPGP1 and AtPGP19.. Mol Biol Cell.

[27] Christie J. M, Swartz T. E, Bogomolni R. A, Briggs W. R (2002). Phototropin LOV domains exhibit distinct roles in regulating photoreceptor function.. Plant J.

[28] Nühse T. S, Stensballe A, Jensen O. N, Peck S. C (2004). Phosphoproteomics of the *Arabidopsis* plasma membrane and a new phosphorylation site database.. Plant Cell.

[29] Salomon M, Zacherl M, Rudiger W (1997). Asymmetric, blue light-dependent phosphorylation of a 116-kilodalton plasma membrane protein can be correlated with the first- and second-positive phototropic curvature of oat coleoptiles.. Plant Physiol.

[30] Friml J, Vieten A, Sauer M, Weijers D, Schwarz H (2003). Efflux-dependent auxin gradients establish the apical-basal axis of *Arabidopsis.*. Nature.

[31] Petrásek J, Friml J (2009). Auxin transport routes in plant development.. Development.

[32] Went F. W, Thimann K. V (1937). Phytohormones..

[33] Franssen J. M, Bruinsma J (1981). Relationships between xanthoxin, phototropism, and elongation growth in the sunflower seedling *Helianthus annuus* L.. Planta.

[34] Shen-Millar J, Cooper P, Gordon S. A (1969). Phototropism and photoinhibition of basipolar transport of auxin in oat coleoptiles.. Plant Physiol.

[35] Cosgrove D. J (1985). Kinetic separation of phototropism from blue-light inhibition of stem elongation.. Photochem Photobiol.

[36] Kaiserli E, Sullivan S, Jones M. A, Feeney K. A, Christie J. M (2009). Domain swapping to assess the mechanistic basis of *Arabidopsis* phototropin 1 receptor kinase activation and endocytosis by blue light.. Plant Cell.

[37] Sullivan S, Thomson C. E, Kaiserli E, Christie J. M (2009). Interaction specificity of *Arabidopsis* 14-3-3 proteins with phototropin receptor kinases.. FEBS Lett.

[38] Motchoulski A, Liscum E (1999). *Arabidopsis* NPH3: a NPH1 photoreceptor-interacting protein essential for phototropism.. Science.

[39] Pedmale U. V, Liscum E (2007). Regulation of phototropic signaling in *Arabidopsis* via phosphorylation state changes in the phototropin 1-interacting protein NPH3.. J Biol Chem.

[40] Lariguet P, Schepens I, Hodgson D, Pedmale U. V, Trevisan M (2006). PHYTOCHROME KINASE SUBSTRATE 1 is a phototropin 1 binding protein required for phototropism.. Proc Natl Acad Sci U S A.

[41] de Carbonnel M, Davis P, Roelfsema M. R, Inoue S, Schepens I (2010). The *Arabidopsis* PHYTOCHROME KINASE SUBSTRATE2 protein is a phototropin signaling element that regulates leaf flattening and leaf positioning.. Plant Physiol.

[42] Sullivan S, Thomson C. E, Lamont D. J, Jones M. A, Christie J. M (2008). *In vivo* phosphorylation site mapping and functional characterization of *Arabidopsis* phototropin 1.. Mol Plant.

[43] Kim J. I, Sharkhuu A, Jin J. B, Li P, Jeong J. C (2007). *yucca6*, a dominant mutation in *Arabidopsis,* affects auxin accumulation and auxin-related phenotypes.. Plant Physiol.

[44] Geisler M, Blakeslee J. J, Bouchard R, Lee O. R, Vincenzetti V (2005). Cellular efflux of auxin catalyzed by the *Arabidopsis* MDR/PGP transporter AtPGP1.. Plant J.

[45] Yang H, Murphy A. S (2009). Functional expression and characterization of *Arabidopsis* ABCB, AUX 1 and PIN auxin transporters in *Schizosaccharomyces pombe*.. Plant J.

[46] Mullet J. E, Klein P. G, Klein R. R (1990). Chlorophyll regulates accumulation of the plastid-encoded chlorophyll apoproteins CP43 and D1 by increasing apoprotein stability.. Proc Natl Acad Sci U S A.

