# Supplementary figures and images for: phot1 Inhibition of ABCB19 Primes Lateral Auxin Fluxes in the Shoot Apex Required For Phototropism
Source: PLoS Biol. 2011 Jun 7;9(6):e1001076. doi: 10.1371/journal.pbio.1001076 (PMC3110179; doi:10.1371/journal.pbio.1001076)

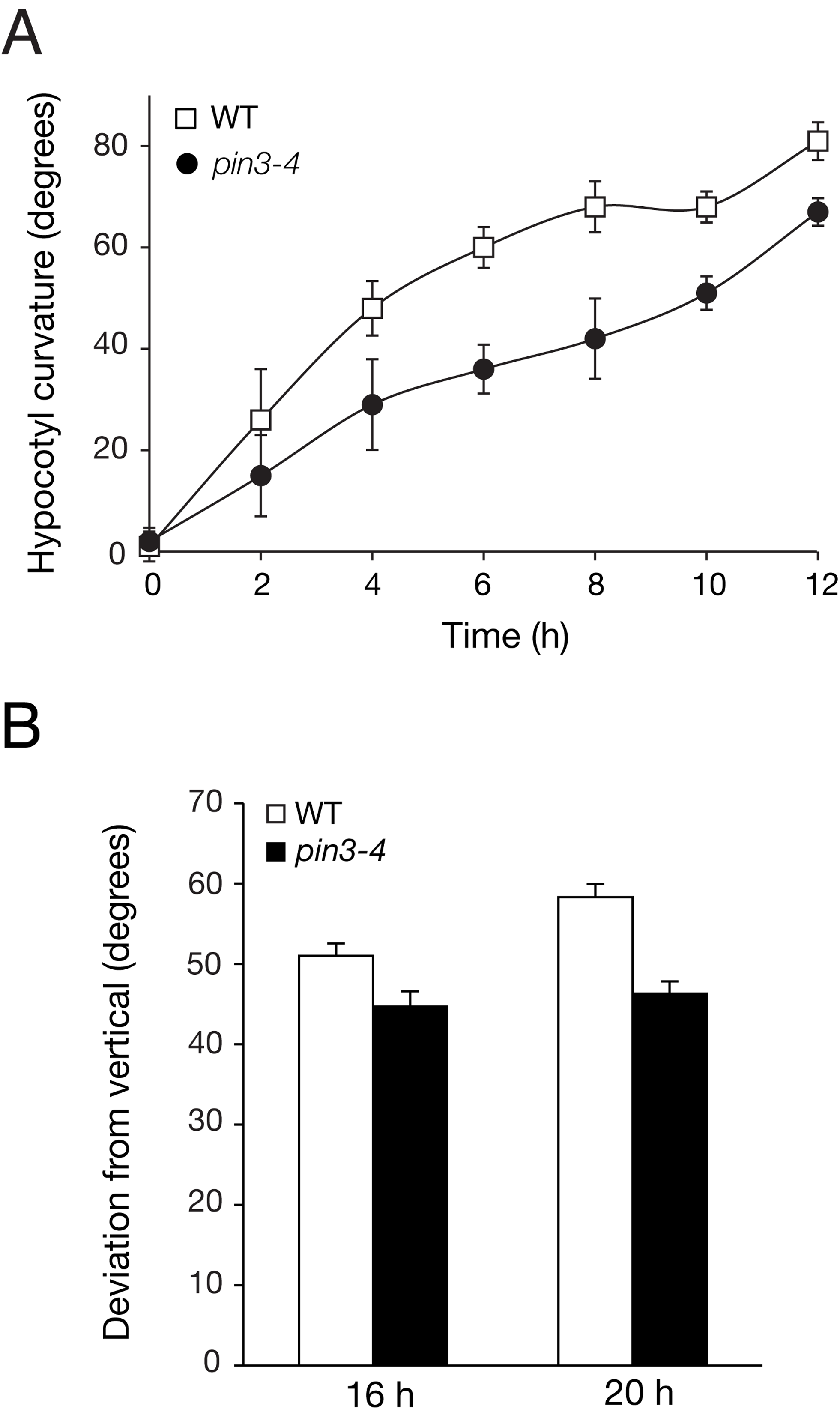

Supplement: Figure S1 — Phototropic responsiveness of pin3 mutants. (A) Phototropic response of 3-d-old etiolated wild-type (WT) and pin3-4 seedlings. Directional blue light (1 µmol m−2 s−1) was supplied for 12 h. Results represent the mean ± standard error, n = 10 seedlings. (B) Phototropic response of dark-acclimated wild-type and pin3-4 seedlings. Seedlings were subjected to directional blue light (1 µmol m−2 s−1) for the times indicated. Results represent the mean + standard error, n = 10 seedlings. (TIF) [file pbio.1001076.s001.tif]

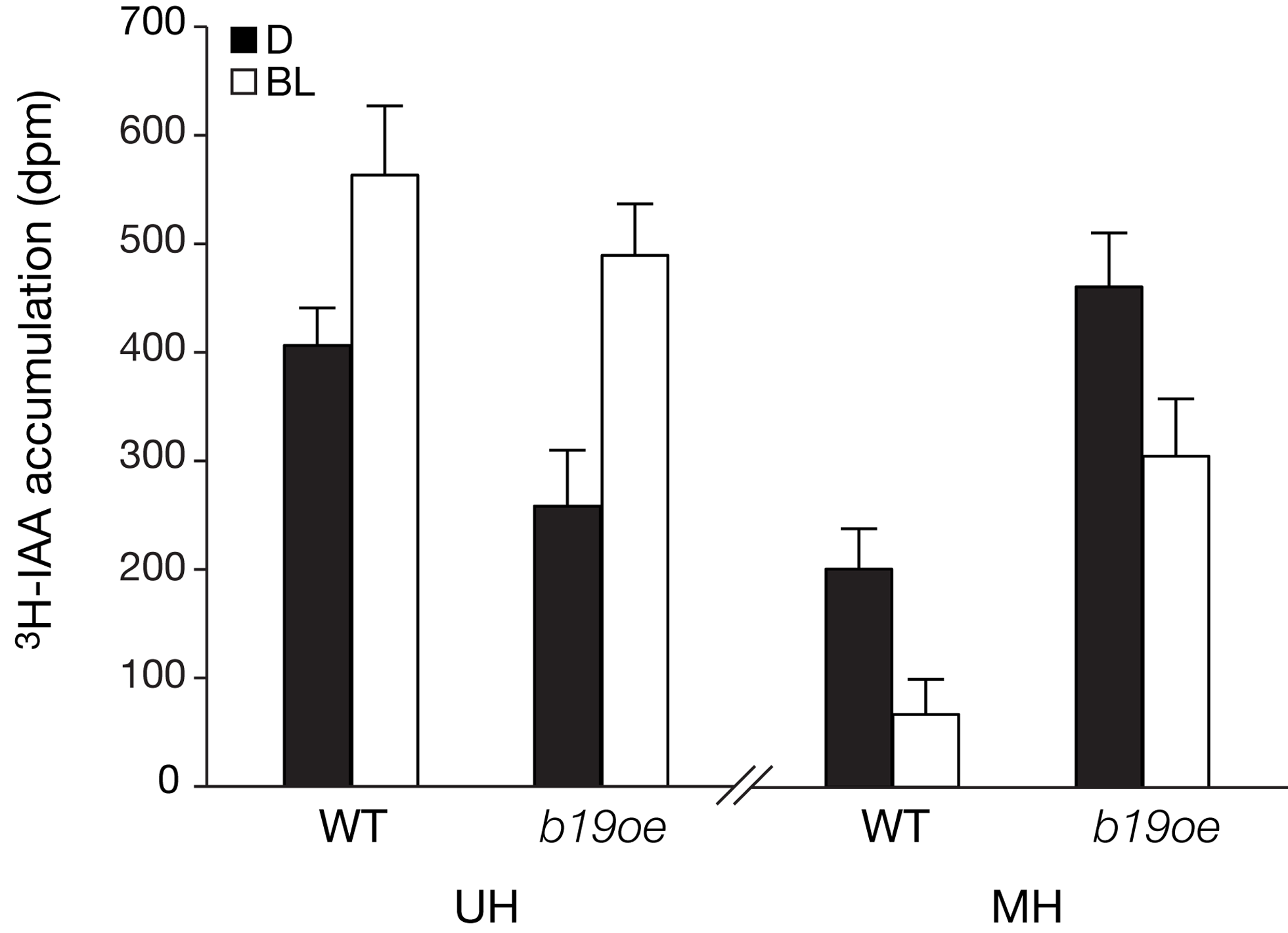

Supplement: Figure S2 — 3H-IAA accumulation in dark-acclimated seedlings overexpressing B19 ( b19oe ). Seedlings were exposed to directional blue light (BL) (1 µmol m−2 s−1) or continued darkness (D). Upper hypocotyls including the cotyledonary node (UH) and mid hypocotyls including the elongation zone (MH) were excised after 2.5 h. Results represent the mean + SD, n = 10 seedlings in three independent experiments. (TIF) [file pbio.1001076.s002.tif]

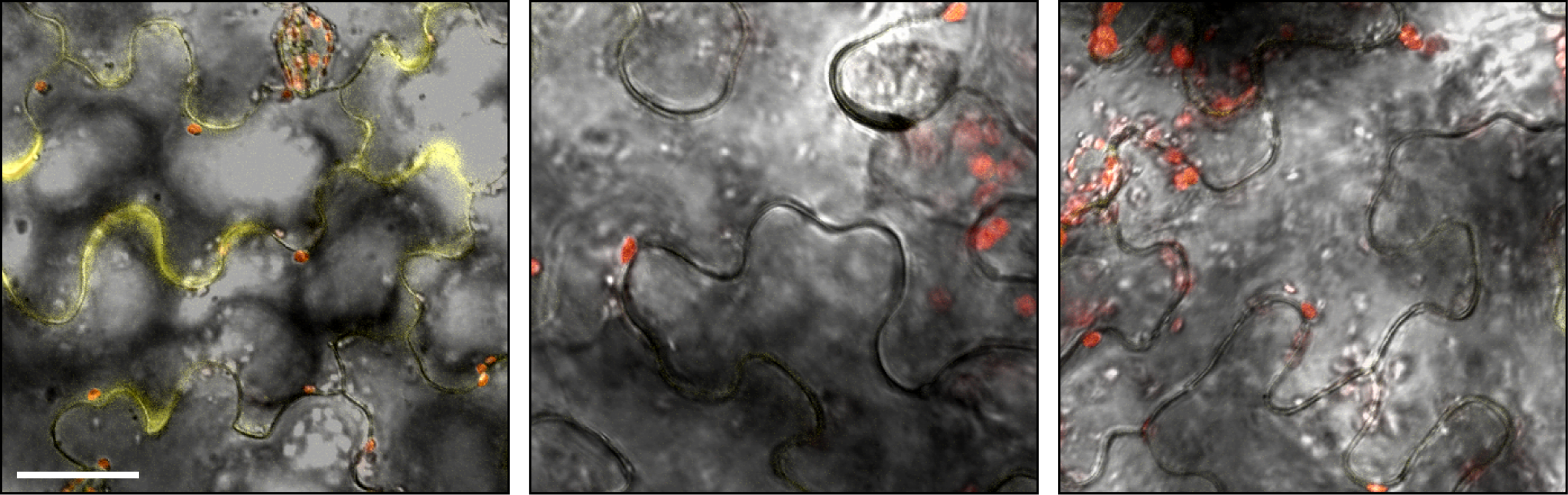

Supplement: Figure S3 — Bright-field BiFC fluorescence images of phot1-YN and B19-YC co-expressed in tobacco epidermal cells. Reconstitution of YFP fluorescence between phot1-YN and B19-YC was visible at the plasma membrane (left). Only background YFP signals were detected for phot1-YN and YC (center) or YN and B19-YC (right). Scale bar = 20 µm and applies to all images. (TIF) [file pbio.1001076.s003.tif]

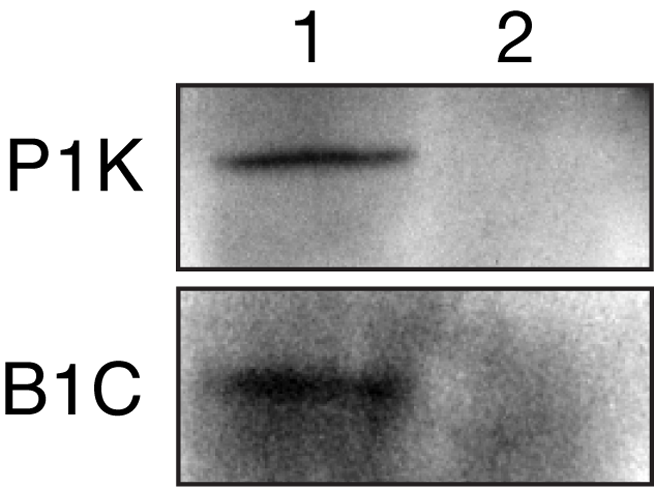

Supplement: Figure S4 — Expression of phot1 kinase and the C-terminal NBD of B1 in yeast. Immunoblot analysis of yeast co-expressing phot1 kinase (P1K) and the C-terminal NBD of B1 (B1C). Protein extracts (10 µg) co-expressing phot1 kinase and either B1C (1) or empty vector controls (2) were probed with anti-GAL4 DNA-binding domain and activation domain antibodies to discriminate phot1 kinase and C-terminal B1 proteins, respectively. (TIF) [file pbio.1001076.s004.tif]

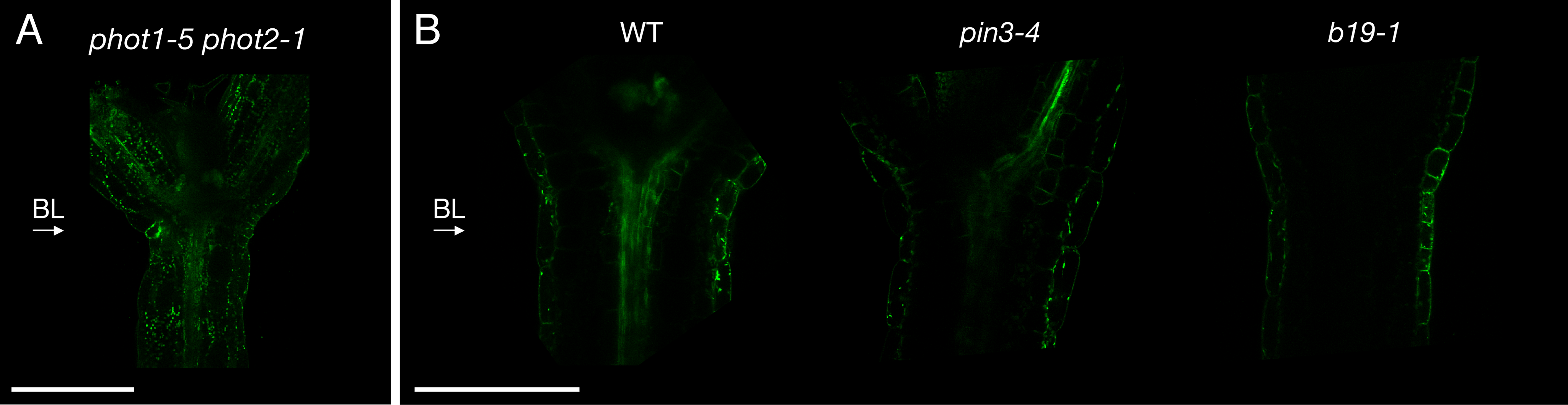

Supplement: Figure S5 — Auxin accumulation in the upper hypocotyl/cotyledonary node of dark-acclimated hypocotyls. (A) DR5rev:GFP signals in phot1–5 phot2–1 seedlings exposed to directional blue light (BL, 1 µmol m−2 s−1) for 3 h. Signal intensity of DR5rev:GFP was noticeably lower in comparison to the other lines examined, giving rise to higher background plastid autofluorescence (represented by spots). (B) DR5rev:GFP signals in seedlings exposed to directional blue light for 12 h. Note the lack of signal in the vascular bundle in b19–1 seedlings and the reduced signal in the vascular bundle below the upper hypocotyl in pin3–4 seedlings. Data are representative of n>5. In each case, scale bar = 200 µm. (TIF) [file pbio.1001076.s005.tif]

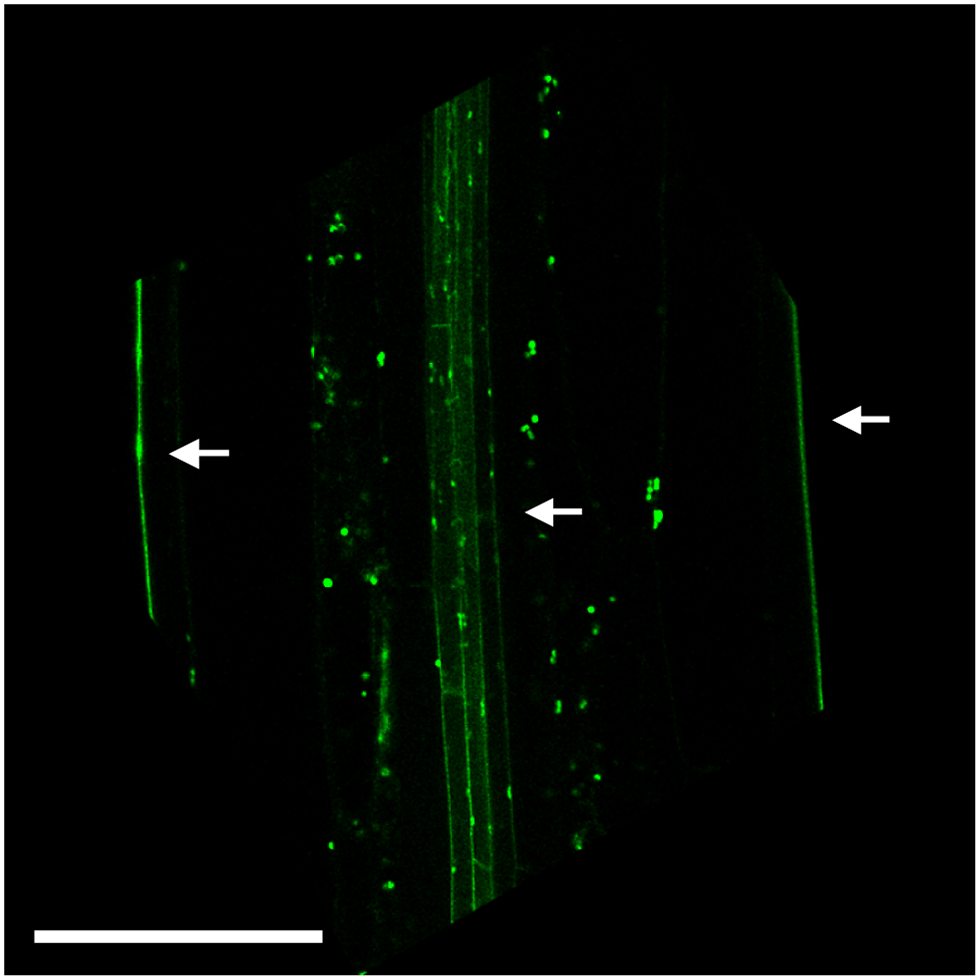

Supplement: Figure S6 — B19-GFP localization in dark-acclimated seedlings. Functional B19:B19-GFP fluorescence is restricted to the central vasculature and epidermis (white arrows). Central spots represent plastid autofluorescence. Data are representative of n>20. Scale bar = 100 µm. (TIF) [file pbio.1001076.s006.tif]

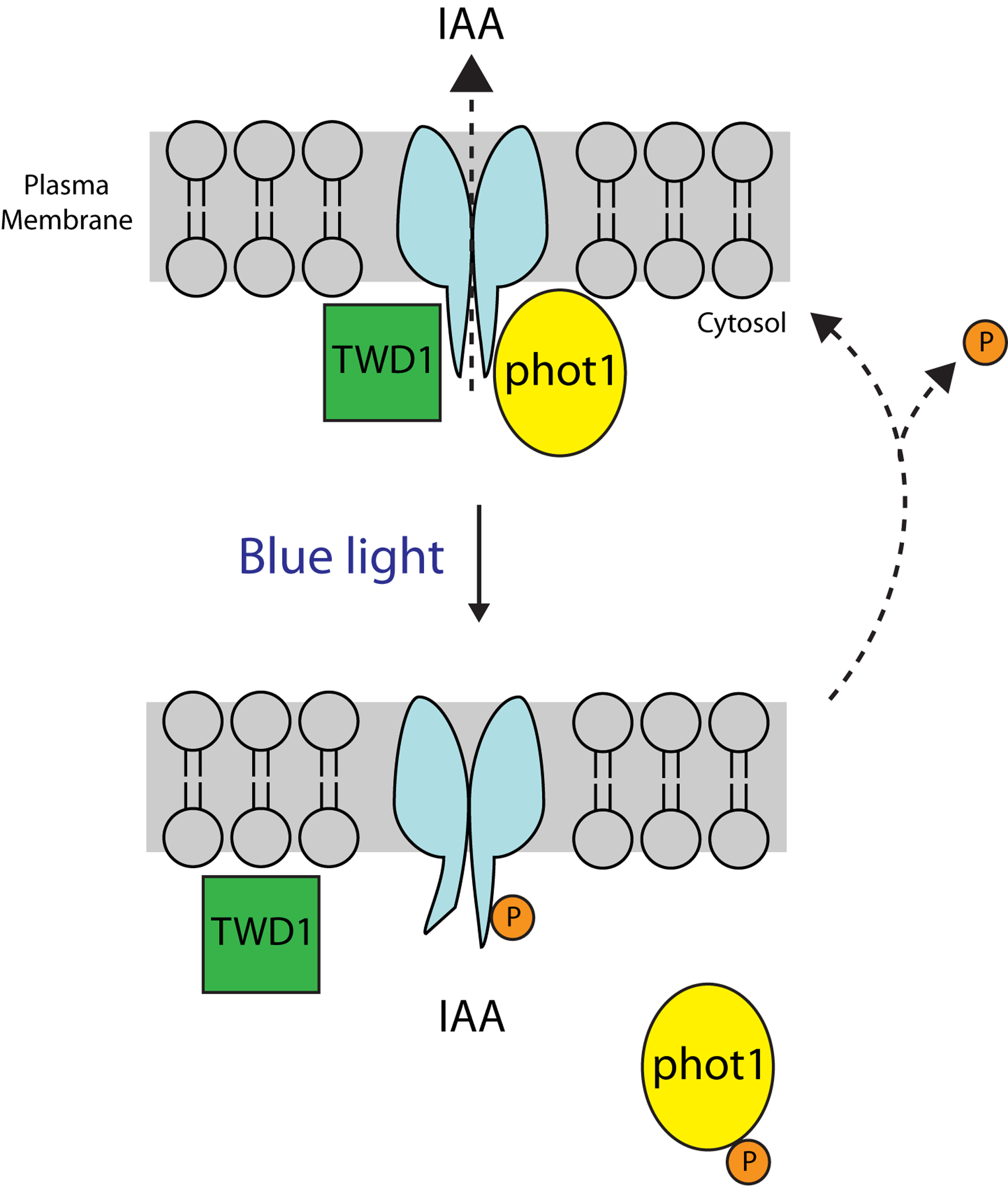

Supplement: Figure S7 — Model depicting a potential mechanism for the blue-light-dependent inhibition of B19-mediated auxin transport activity. In darkness or ground state conditions, B19 (blue, center) actively exports IAA from the cytosol via interactions with its positive regulator TWD1. Under these conditions, B19 also interacts with the blue-light photoreceptor phot1. In response to blue-light exposure, phot1 undergoes autophosphorylation and transphosphorylates B19. Sites of B19 phosphorylation may also be targets for other regulatory kinases. Phosphorylation of B19 may promote an alteration in protein structure that disrupts its interaction with TWD1, leading to an inhibition of IAA efflux. phot1 is internalized upon receptor autophosphorylation, creating an inhibitory mechanism that is transient and becomes reactivated upon dephosphorylation of B19 and phot1 by as yet unidentified protein phosphatases. (TIF) [file pbio.1001076.s007.tif]
