# Supplementary material for: phot1 Inhibition of ABCB19 Primes Lateral Auxin Fluxes in the Shoot Apex Required For Phototropism
Source: PLoS Biol. 2011 Jun 7;9(6):e1001076. doi: 10.1371/journal.pbio.1001076 (PMC3110179; doi:10.1371/journal.pbio.1001076)
Supplement: Table S1 — Liquid chromatography–tandem mass spectrometry analysis of phot1-GFP immunoprecipitates. Proteins identified with a Mascot score >100 are shown. aPHOT1:phot1-GFP was immunoprecipitated from 3-d-old etiolated Arabidopsis seedlings kept in darkness (D) or exposed to a blue light (BL, 20 µmol m−2 s−1) for 5 min. bTAIR AGI numbers given where mass spectrometry data match a single accession; “various” denotes multiple isoforms were identified. (DOC) [file pbio.1001076.s008.doc]

| **Sample^a^** | **Protein Name** | **Function** | **AGI Number^b^** |
| --- | --- | --- | --- |
| D | ABCB19 (ABC transporter B family member 19) | Auxin transport | At3g28860 |
| D | AtBAG7 (BCL-2-associated athanogene 7) | Calmodulin binding | At5g62390 |
| D | CRU3 (CRUCIFERIN 3) | Nutrient reservoir activity | At4g28520 |
| D | ATPase 1, plasma membrane-type | Proton transport | At2g18960 |
| D | PIP1B (Plasma membrane intrinsic protein 1B) | Water channel activity | At2g45960 |
| BL | S-adenosyl-L-homocysteine hydrolase | Adenosylhomocysteinase activity | At4g13940 |
| BL | EIF4A (eukaryotic translation initiation factor 4A) | ATP-dependent helicase activity |  |
| BL | Jacalin lectin family protein | Copper ion binding | At3g16460 |
| BL | Actin | Cytoskeleton |  |
| BL | Glyceraldehyde-3-phosphate dehydrogenase | Glycolysis, reactive oxygen signalling | At3g04120 |
| BL | RPT2 | Protein binding, phototropic signalling | At2g30520 |
| D and BL | Heat shock cognate 70kDa protein 1 | ATP binding, protein folding | At5g02500 |
| D and BL | PYK10 | Beta-glucosidase activity | At3g09260 |
| D and BL | Kinesin light chain-related | Binding | At4g10840 |
| D and BL | Phototropin 1 | Blue light receptor | At3g45780 |
| D and BL | Tubulin alpha chain | Cytoskeleton | various |
| D and BL | Tubulin beta chain |  | various |
| D and BL | Dynamin-like proteins | GTP binding, vesicle trafficking | various |
| D and BL | Clathrin heavy chain | Intracellular protein transport | At3g11130 |
| D and BL | AtCIMS (cobalamin-independent methionine synthase) | Methionine biosynthesis | At5g17920 |
| D and BL | Cupin family protein | Nutrient reservoir activity | At4g36700 |
| D and BL | ATPase 2, plasma membrane-type | Proton transport | At4g30190 |
| D and L | 40S ribosomal proteins | Structural constituent of ribosome | various |
| D and L | 60S ribosomal proteins |  | various |
| D and BL | PIP3 (Plasma membrane intrinsic protein 3) | Water channel activity | At4g35100 |
