# Supplementary material for: phot1 Inhibition of ABCB19 Primes Lateral Auxin Fluxes in the Shoot Apex Required For Phototropism
Source: PLoS Biol. 2011 Jun 7;9(6):e1001076. doi: 10.1371/journal.pbio.1001076 (PMC3110179; doi:10.1371/journal.pbio.1001076)
Supplement: Table S2 — Liquid chromatography–matrix-assisted laser desorption/ionization mass spectrometry analysis of B19 immunoprecipitates. Proteins identified with a Mascot score >150 are shown. TAIR AGI numbers are given. B19:B19-HA was immunoprecipitated from 5-d-old Arabidopsis seedlings. aPreviously identified in B19 fractions [12]. (DOC) [file pbio.1001076.s009.doc]

| **Protein Name** | **Function** | **AGI Number** |
| --- | --- | --- |
| ATPase 2, plasma membrane-type | Proton transport | At4g30190^a^ |
| Phot1 (Phototropin 1) | Blue light receptor | [At3g45780](http://proteins.wikiprofessional.org/index.php/Term:At3g45780) |
| PIN1 (PIN-FORMED 1) | Auxin transport | At1g73590^a^ |
| Heat shock cognate 70kDa protein | ATP binding, protein folding | At5g02500 |
| Heat shock cognate 70kDa protein | ATP binding, protein folding | At4g16650^a^ |
| Glyceraldehyde-3-phosphate dehydrogenase | Glycolysis, reactive oxygen signalling | At3g04120 |
| FLA11 (Fasciclin-like arabinogalactan protein 11) | Cell surface adhesion protein | [At5g03170](http://www.wikiproteins.org/index.php/Term:At5g03170)^a^ |
| APL1/GDSL lipase | Lipase/hydrolase | At5g03610^a^ |
| Glycosyl hydrolase 3 | Hydrolase | [At5g04885](http://www.arabidopsis.org/servlets/TairObject?type=gene&id=500439049) |
