# Supplementary material for: phot1 Inhibition of ABCB19 Primes Lateral Auxin Fluxes in the Shoot Apex Required For Phototropism
Source: PLoS Biol. 2011 Jun 7;9(6):e1001076. doi: 10.1371/journal.pbio.1001076 (PMC3110179; doi:10.1371/journal.pbio.1001076)
Supplement: Table S3 — Lines used in this study. (DOC) [file pbio.1001076.s010.doc]

| **Line** | **AGI Number** | **Source** |
| --- | --- | --- |
| *aux1-7* | AT2G38120 | Swarup R, Kargul J, Marchant A, Zadk D, Rahman A, et al. (2004) Plant Cell 16: 3069-3083. |
| *aux1 lax2 lax3* | AT2G38120 AT2G21050 AT1G77690 | Swarup K, Benková E, Swarup R,  Casimiro I, Péret B, et al. (2008) Nat Cell Biol 10: 946-954. |
| *b1-1*  *b19-1* | AT2G36910  AT3G28860 | Noh B, Bandyopadhyay A, Peer W. A, Spalding E. P, Murphy, A. S (2003) Nature 423: 999-1002. |
| *b4-1* | AT2G47000 | Terasaka, K, Blakeslee J. J, Titapiwatanakun B, Peer W. A, Bandyopadhyay A, et al. (2005) Plant Cell 17: 2922-2939. |
| *b1-1 b4-1 b19-1* |  | This work |
| *pin1-1* | AT1G73590 | Müller A, Guan C, Gälweiler L, Tänzler P., Huijser P, et al. (1998) EMBO J 17: 6903-6911. |
| *pin1-5* | AT1G73590 | Smyth, D. R., Monash University, Melbourne Australia |
| *pin2 (agr1-1)* | AT5G57090 | Chen R, Hilson P, Sedbrook J, Rosen E, Caspar T, et al. (1998) Proc Natl Acad Sci USA 95: 15112-15117. |
| *pin2 (eir1-1)* | AT5G57090 | Luschnig C, Gaxiola R. A, Grisafi P, Fink G. R (1998) Genes Dev 12: 2175-2187. |
| *pin3-4*  *pin4-1* | AT1G70940  AT2G01420 | Friml J, Wiśniewska J, Benková E, Mendgen K, Palme K (2002) Nature 415: 806-809. |
| *pin7* (N548791) | AT1G23080 | NASC, this work |
| *pin1-5 pin3-4* |  | This work |
| *pin3-4 b19-1* |  | This work |
| *pin1-1 b1-1 b19-1*  *B19*:B19-GFP |  | Blakeslee J. J, Bandyopadhyay A, Lee O. R, Mravec J, Titapiwatanakun B, et al. (2007) Plant Cell 19: 131-147. |
| *phot1-5*  *phot2-1*  *phot1-5 phot2-1* | AT3G45780  AT5G58140 | Kinoshita T, Doi M, Suetsugu N, Kagawa T, Wada M, et al. (2001) Nature 414: 656-660. |
| *B19*:B19-HA |  | Geisler M, Blakeslee J. J, Bouchard R, Lee O. R, Vincenzetti V, et al. (2005) Plant J 44: 179-194. |
| *DR5rev*:GFP |  | Friml J, Vieten A, Sauer M, Weijers D, Schwarz H, et al. (2003) Nature 426: 147-153. |
| *PIN3*:PIN3-GFP |  | Zádníková P. J, Petrásek J, Marhavy P, Raz V, Vandenbussche F, et al. (2010) Development 137: 607-617. |
| *PHOT1*:phot1-GFP |  | Sakamoto K, Briggs W. R (2002) Plant Cell 14: 1723-1735. |
| *35S*:GFP-Lti6b |  | Cutler S. R, Ehrhardt D. W, Griffitts J. S, Somerville C. R (2000) Proc Natl Acad Sci USA 97: 3718-3723. |
| *cry1-304 cry2-1* | AT4G08920  AT1G04400 | Lascève G, Leymarie J, Olney, M. A, Liscum E, Christie J. M, et al. (1999) Plant Physiol 120: 605-614 . |
| *phyA-201 phyB-5* | AT1G09570  AT2G18790 | Poppe C, Schäfer E (1997) Plant Physiol 114: 1487-1492. |
